# Retrieval-Augmented Large Language Model for Angiographic Prediction of Coronary Physiology

**DOI:** 10.3390/jcm15135253

**Published:** 2026-07-05

**Authors:** Sant Kumar, Keshav Nandakumar, Pedro A. Villablanca, Vikas Aggarwal, Hursh Naik, Nezar Falluji

**Affiliations:** 1 School of Medicine, Creighton University, Phoenix, AZ 85012, USA; 2 Department of Cardiovascular Medicine, St. Joseph Hospital and Medical Center, 500 West Thomas Road, Phoenix, AZ 85013, USA; 3 Division of Cardiovascular Medicine, Henry Ford Hospital, Detroit, MI 48202, USA

**Keywords:** coronary angiography, retrieval augmented generation, instantaneous wave-free ratio, artificial intelligence, coronary physiology

## Abstract

**Background:** Invasive physiologic assessment is often needed for moderately stenotic coronary lesions, although it remains underused in routine practice. It remains unknown whether GPT-based large language models (LLMs) can estimate coronary physiology from coronary angiographic images, and whether retrieval augmentation can improve this task. **Methods:** We performed a retrospective pilot study of consecutive cases undergoing coronary angiography with invasive instantaneous wave-free ratio (iFR) assessment between 2023 and 2025. Eligible cases required invasive iFR and two orthogonal end-diastolic still frames of the target vessel at maximal opacification. We compared a baseline GPT-5.2 model without retrieval-augmented generation (RAG), termed No-RAG, with the same GPT-5.2 model using RAG, termed RAG. Both conditions received identical angiographic frames and structured clinical text. The RAG modification added the top five case-specific text chunks retrieved from four coronary physiology/revascularization documents to provide physiologic thresholds, guideline context, and uncertainty framing; no additional angiographic images or lesion-specific iFR information were provided. Frame-level predictions were averaged to derive a case-level predicted iFR. The primary endpoint was agreement between predicted and measured iFR. **Results:** Of 34 eligible cases screened, 32 vessels were included. The cohort comprised 25/32 cases (78.1%) with significant disease (iFR ≤ 0.89) and 7 cases classified as non-ischemic. Without RAG, mean absolute error (MAE) was 0.064 and the root mean square error (RMSE) was 0.083, with weak correlation with invasive iFR (r = 0.205, *p* = 0.259). With RAG, point estimates favored improved continuous agreement, with MAE decreasing to 0.029, RMSE to 0.038, and correlation increasing to r = 0.830 (*p* < 0.001). Threshold-based classification also yielded higher point estimates for accuracy, increasing from 0.750 to 0.906. **Conclusions:** In this small pilot study, improved point estimates for agreement between LLM-predicted and invasively measured iFR were seen after adding RAG to a GPT-based model for estimating iFR from angiographic imaging. These findings suggest that functionally classifying coronary stenoses is limited by overestimating severity in less severe stenoses, and that a scaling correction is needed. The results, however, require validation in larger, more balanced cohorts.

## 1. Introduction

Large language models (LLMs) are increasingly being used in medicine for tasks such as clinical summarization, decision support, literature synthesis, and image-informed interpretation [1,2]. In cardiovascular medicine, these systems have attracted particular interest because they can rapidly integrate large volumes of clinical and imaging data and generate structured recommendations [3]. However, their optimal role remains unclear. Although LLMs may improve efficiency and consistency, it is uncertain when they provide meaningful clinical benefit, whether their reasoning aligns with evidence-based practice, and how best to augment their outputs to reduce overreliance on superficial anatomic or pattern-based interpretation [1].

In percutaneous coronary intervention (PCI), invasive physiologic assessment remains a key tool for determining the hemodynamic significance of angiographically intermediate lesions and for guiding revascularization decisions [4,5]. Both fractional flow reserve (FFR) and nonhyperemic pressure ratios such as instantaneous wave-free ratio (iFR) have helped move practice beyond angiographic appearance alone, recognizing that anatomic stenosis severity does not always reflect functional ischemia [5,6]. At the same time, physiology remains underused in routine practice because it requires additional equipment, wire manipulation, expertise, procedural time, and cost and may be limited by workflow barriers, operator preference, and selective use in higher-yield or uncertain lesions [7,8]. As a result, real-world management may still be influenced heavily by angiographic morphology, even when physiology could provide a more appropriate decision framework.

Against this background, retrieval-augmented generation (RAG) [9] may improve the clinical usefulness of LLMs by pairing the angiogram with retrieved text on coronary physiology, decision thresholds, and interpretive context, rather than relying only on the model’s unaided response. RAG integrates LLMs with external knowledge bases to enable real-time access to documents like scientific consensus statements [10]. It uses a retriever to find information and a generator to synthesize responses [11]. Whether this type of augmentation meaningfully changes angiographic interpretation or physiologic prediction has not been well studied. We therefore performed a pilot study of patients undergoing coronary angiography with invasive iFR assessment to compare standard LLM output with retrieval-augmented output, focusing on predicted iFR, threshold-based ischemia classification, and the integration of physiology-guided reasoning.

## 2. Methods

### 2.1. Study Design

This retrospective, exploratory pilot study was conducted at a single hospital center between 2023 and 2025. Consecutive cases undergoing invasive coronary angiography with invasive instantaneous wave-free ratio (iFR) assessment were screened from the institutional catheterization laboratory database. All index procedures and invasive physiologic assessments were performed by a single operator.

Eligible cases required (1) diagnostic coronary angiography of a target coronary lesion, (2) invasive iFR measurement during the index procedure, and (3) 2 orthogonal angiographic views of the target vessel suitable for model-based review. Incomplete angiographic data were defined as the absence of either required orthogonal view for a given vessel. Cases with incomplete angiographic data or insufficient documentation were excluded. No prespecified clinical or anatomic lesion-level exclusion criteria were applied. All lesions with invasive iFR were considered. Specifically, prior CABG, severe diffuse disease, left main or ostial lesion location, chronic total occlusion, heavy calcification, and poor contrast filling were not exclusion criteria unless they prevented identification of the target lesion, selection of the required angiographic views, or adequate documentation. Cases were excluded only for incomplete angiographic data or insufficient documentation.

Clinical, angiographic, physiologic, and procedural data were abstracted from the medical record and final catheterization report. The reference physiologic standard was the invasive iFR measured during the index procedure, with the pressure wire positioned as distally as feasible within the target vessel. For LLM-based assessment, iFR was estimated from angiographic still frames corresponding to the same vessel segment, with the model instructed to approximate physiologic severity based on the distal vessel territory, consistent with the location where invasive iFR is typically measured.

When multiple invasive iFR measurements were available, waveform adjudication was performed to confirm technical adequacy, including appropriate equalization and absence of clinically significant drift when pullback documentation was available. Among technically adequate measurements, the lowest distal target-vessel iFR was selected for the primary analysis. This approach was chosen because the study endpoint was vessel-level physiologic significance, and the lowest distal value reflects the greatest cumulative pressure loss across the interrogated vessel. Measurements obtained at different wire positions were not averaged because doing so could combine distal, segmental, or pullback values that do not represent the same physiologic sampling location.

For the target vessel, 2 orthogonal still frames were selected at end diastole during maximal vessel opacification to reflect routine invasive assessment of lesion severity and morphology. Model-predicted iFR values were generated at the image level and then averaged across the 2 angiographic frames to derive a case-level predicted iFR for each model condition. Full angiographic cine loops were not used because the LLM workflow implemented in this study did not support direct cine-loop input. Still-frame selection was therefore used as a pragmatic image-input strategy rather than as a substitute for complete angiographic review.

Selected end-diastolic angiographic frames were exported from the source angiographic DICOM runs as de-identified raster images in PNG format, with DICOM headers, acquisition metadata, and patient identifiers removed. Images were retained at native resolution when possible, typically 512 × 512 pixels, and images exceeding 1024 pixels on the longest axis were downsampled to a maximum long-axis dimension of 1024 pixels while preserving aspect ratio. No manual cropping, annotation, vessel segmentation, contrast enhancement, edge detection, quantitative coronary angiography, vessel reconstruction, or computational flow processing was performed. The model therefore received only the rasterized still-frame image files and structured text prompt; DICOM cine data, frame timing, acquisition-angle metadata, and pressure-wire data were not passed to the LLM. The identical image files were used in the No-RAG and RAG conditions and were submitted as image inputs through the OpenAI Responses API.

The reference physiologic standard was the clinically recorded invasive iFR measured during the index procedure after pressure-wire equalization, with the pressure sensor positioned as distally as feasible within the target vessel. Available waveform tracings and procedural documentation were reviewed for technical adequacy, including appropriate equalization and absence of clinically significant pressure drift on pullback when pullback documentation was available. When multiple technically adequate iFR measurements were available, the lowest value was selected for analysis. Systematic post hoc drift correction was not performed.

Because cases were identified from patients undergoing invasive iFR assessment rather than from an unselected angiography cohort, lesion inclusion was dependent on the clinical decision to perform physiologic interrogation. This design may preferentially capture lesions considered angiographically intermediate, clinically uncertain, discordant with symptoms or noninvasive testing, or relevant to revascularization decision-making. Conversely, lesions judged clearly nonobstructive, clearly severe, technically unsuitable for pressure-wire assessment, or not clinically actionable may be underrepresented.

The study was conducted in accordance with the principles of the Declaration of Helsinki and good clinical practice. Given the retrospective nature of the analysis, informed consent was waived. The study protocol was approved by the institutional review board.

### 2.2. Large Language Model Evaluation

Each case was evaluated under 2 conditions: a standard non-retrieval condition (No-RAG) and a retrieval-augmented generation condition (RAG). In both conditions, the model received the same 2 orthogonal end-diastolic still frames obtained at maximal vessel opacification and was prompted to interpret the angiographic case and provide a structured assessment of lesion significance, physiologic severity, and explanatory reasoning. In both conditions, the same structured clinical information was also provided as text within the prompt, organized into 5 domains: demographics, presentation/indication, medical history, laboratory values, and medications. The primary quantitative output was the model-predicted iFR. For threshold-based analyses, a binary ischemia classification was derived using the predefined cutoff of predicted iFR ≤ 0.89 versus >0.89, consistent with the invasive ischemic threshold used in the study and contemporary physiologic guidance [7,8].

To improve reproducibility, the complete system instructions, user prompts, RAG-specific prompt wrapper, output format constraints, and prompt-refinement history are provided in Supplementary Table S1. In brief, the model was instructed to estimate vessel-level iFR from 2 orthogonal still angiographic frames and structured clinical data, to provide frame-level and averaged case-level predicted iFR values, and to classify ischemia using the prespecified iFR threshold of ≤0.89. The RAG condition used the identical case prompt with the addition of the top 5 retrieved coronary physiology/revascularization text chunks.

For output generation, the model used was OpenAI GPT-5.2, accessed via the OpenAI API with the gpt-5.2-2025-12-11 snapshot identifier. GPT-5.2 was released by OpenAI on 11 December 2025. Inference was performed in a Google Colaboratory Python (V 3.12) environment using the OpenAI Responses API endpoint, v1/responses. Model parameters were temperature 0.2, top-*p* 0.98, and medium verbosity. No random seed was set, and no deterministic decoding mode was used. Each case was evaluated once per condition and per angiographic frame; therefore, run-to-run reproducibility was not formally assessed. Accordingly, the analysis reflects single-run model output under the specified settings rather than averaged or ensemble-based repeated sampling. In the No-RAG condition, outputs were generated using the case input alone. In the RAG condition, the same case input was supplemented by retrieved external reference material intended to ground the model’s interpretation in coronary physiology, physiologic thresholds, and revascularization decision-making.

This study was reported in accordance with the TRIPOD-LLM checklist for studies evaluating large language models. A completed checklist with item-by-item manuscript cross-references is provided in Supplementary Table S2. The intended use setting for this pilot study was retrospective research evaluation of LLM-generated iFR estimates from angiographic still frames and structured clinical text. The intended users are cardiology investigators and, in future validation studies, cardiologists or interventional cardiologists evaluating whether LLM outputs align with invasive physiology. The model was not evaluated as an autonomous diagnostic tool, was not used for patient care, and was not intended to replace invasive physiologic assessment or clinician judgment.

### 2.3. Retrieval-Augmented Generation

For the retrieval-augmented condition, the following documents were combined into a single PDF document and uploaded into Google Colaboratory: (1) 2023 AHA/ACC/ACCP/ASPC/NLA/PCNA Guideline for the Management of Patients With Chronic Coronary Disease [9], (2) 2025 ACC/AHA/ACEP/NAEMSP/SCAI Guideline for the Management of Patients With Acute Coronary Syndromes [8], (3) 2021 ACC/AHA/SCAI Guideline for Coronary Artery Revascularization [7], and (4) Evidence-Based Practices in the Cardiac Catheterization Laboratory: Invasive Epicardial Coronary Physiologic Assessment: A Scientific Statement From the American Heart Association [10]. These sources were selected to provide physiology-oriented decision thresholds, interpretive context, and uncertainty framing.

The combined PDF was loaded using PyPDFLoader, and the extracted text was split into 500-character chunks with a 100-character overlap. Text chunks were embedded using the OpenAI text-embedding-3-small model, producing 1536-dimensional embeddings. The FAISS dense-vector index was created from these embeddings. Retrieval was performed using a top-k nearest-neighbor dense retriever with k = 5. Similarity was calculated using cosine similarity, implemented as an inner-product search over L2-normalized embeddings. No BM25 retrieval, hybrid retrieval, maximal marginal relevance filtering, cross-encoder reranking, manual reranking, or LLM-based reranking was performed. Chunk retrieval was performed separately for each case-specific prompt, rather than using a fixed set of chunks across all cases. The retrieval query was generated from the structured case text and task instruction; angiographic images themselves were not embedded for retrieval. Thus, retrieval was case-specific through the text prompt but not through image-vector search. The text was split into 500-character chunks with a 100-character overlap. This chunk size was selected pragmatically to preserve short guideline- and physiology-relevant passages while limiting retrieval of overly long, nonspecific text segments. The top-k retrieval value was set to 5 to provide several relevant context passages without substantially increasing prompt length or introducing excessive off-target material. These retrieval parameters were specified before model inference and were not tuned based on measured iFR values, classification labels, mean absolute error (MAE), area under curve (AUC), or other performance results. No formal sensitivity analysis varying chunk size, overlap, or k value was performed. The RAG model output was then generated using the retrieved information from the top 5 most relevant document chunks. This retrieval pipeline was intended to provide contextually relevant physiologic and evidence-based material to supplement angiographic interpretation, not to replace the angiographic input or to provide patient-specific lesion labels. Any influence on predicted iFR would therefore be expected to occur indirectly through reasoning calibration, threshold framing, and handling of uncertainty.

### 2.4. Output Review and Abstraction

Model outputs were reviewed for both quantitative and qualitative analysis. For quantitative analyses, vessel-level predicted iFR values and derived ischemia classifications were extracted from each model output. For qualitative analyses, No-RAG and RAG outputs were reviewed in parallel to identify recurrent terminology and reasoning patterns related to lesion morphology, physiology, clinical context, and interpretive framing. Particular attention was paid to whether outputs transitioned directly from angiographic morphology to action-oriented procedural planning or instead incorporated physiology-guided thresholds, evidence-based language, and explicit uncertainty framing before estimating physiologic significance.

Model outputs were de-identified and assigned neutral condition labels before review. Two independent reviewers, blinded to whether outputs were generated under the No-RAG or RAG condition, extracted predicted iFR values and coded qualitative output features using predefined domains. Discrepancies were resolved by consensus. Model condition was unblinded only after quantitative abstraction and qualitative coding were completed.

### 2.5. Study Endpoints

The primary endpoint was agreement between model-predicted iFR and measured invasive iFR. Secondary endpoints included ischemia classification performance using invasive iFR ≤ 0.89 as the reference standard and qualitative differences in reasoning patterns between No-RAG and RAG outputs. iFR was used as the reference standard because it was the routine invasive physiologic index used in clinical practice at our institution during the study period. FFR was not the default institutional workflow for these cases. Therefore, the present analysis specifically evaluates alignment with clinically measured iFR, and the findings should not be assumed to generalize directly to FFR without separate validation using FFR-labeled cases. Exploratory analyses examined shifts in lesion description and interpretive framing, including mentions of CABG, lesion complexity descriptors, and the frequency of IVUS recommendations.

### 2.6. Statistical Analysis

All analyses were conducted at the vessel level. The primary quantitative endpoint was agreement between model-predicted and measured invasive iFR. When multiple invasive iFR measurements were available, waveform adjudication was performed to assess signal quality, including appropriate pressure-wire equalization and absence of drift on pullback. Among measurements deemed technically adequate, the lowest value was selected for analysis. Predicted iFR values were averaged across angiographic images per patient. Agreement and calibration between model-predicted and measured iFR were assessed using MAE, root mean square error (RMSE), Bland–Altman bias and 95% limits of agreement, calibration-in-the-large, calibration slope, and Lin’s concordance correlation coefficient. Calibration-in-the-large was defined as the mean prediction error, calculated as the difference between predicted and measured iFR. The calibration slope was estimated using a linear calibration model that compared predicted and measured iFR, with perfect calibration corresponding to a slope of 1.0. Pearson correlation was calculated only as a secondary measure of linear association. For ischemia classification, an exploratory Brier score was calculated using the binary predicted ischemia label (iFR ≤ 0.89 vs. >0.89). Because the model did not generate calibrated probabilities of ischemia, this was interpreted as a hard-label squared-error score rather than a probability-calibration metric. Decision-curve analysis was not performed because the model outputs were numeric iFR estimates and threshold-derived binary classifications rather than calibrated treatment probabilities or prespecified clinical utility estimates.

Ischemia classification performance was evaluated using invasive iFR ≤ 0.89 as the reference standard. Threshold-dependent metrics (sensitivity, specificity, PPV, NPV, accuracy) and threshold-independent discrimination (area under the receiver operating characteristic curve [AUC]) were calculated. A supplemental sensitivity analysis translated the predicted iFR into a binary PCI recommendation and compared it with the operator’s management, as documented in the final catheterization report, using the same threshold-based metrics and the McNemar exact test.

Uncertainty was estimated using nonparametric bootstrap resampling (15,000 iterations) to derive percentile-based 95% confidence intervals for AUC, MAE, and between-model differences (RAG − No-RAG). Patient-level changes in absolute error (|error_RAG| − |error_NoRAG|) were calculated and visualized using waterfall plots. For class-dependent metrics, including AUC and between-model AUC differences, resampling was stratified by invasive iFR classification, with each bootstrap replicate sampling with replacement from the 25 ischemia-positive vessels and 7 ischemia-negative vessels to preserve the observed class distribution. The same resampled vessel indices were applied to both No-RAG and RAG predictions to preserve the paired study design. The 15,000 iterations were used to reduce Monte Carlo variability in the percentile estimates and should not be interpreted as compensating for the limited sample size.

To explore potential mechanisms underlying performance differences, a structured qualitative content analysis of model outputs was performed. All No-RAG and RAG outputs were reviewed in parallel by two independent reviewers. Review focused on four predefined domains: (1) use of physiology-referenced language (e.g., physiology-guided assessment, physiologic thresholds, post-PCI targets); (2) reliance on morphology-driven descriptors (e.g., long diffuse disease, focal high-grade stenosis, early action-oriented escalation); (3) integration of clinical context (e.g., comorbidities, medication status, syndrome classification); and (4) handling of borderline or uncertain cases. Discrepancies were resolved by consensus. Representative excerpts were used to illustrate reasoning patterns. This qualitative analysis was intended to assess whether retrieval augmentation shifted interpretation from morphology-dominant inference toward greater integration of physiologic thresholds, clinical context, and evidence-based decision framing.

A *p*-value ≤ 0.05 was considered statistically significant. Analyses were conducted using R version 4.4.2 (R Core Team, Vienna, Austria) and MedCalc Statistical Software version 12.7.7 (MedCalc Software, Ostend, Belgium).

## 3. Results

A total of 34 cases were reviewed. Following the exclusion of two cases due to incomplete angiographic data (missing at least one of the two required orthogonal views), 32 vessels were included in the final analysis. The prevalence of physiologically significant disease (iFR ≤ 0.89) was 25/32 cases (78.1%). PCI was performed by the operator in 28 of 32 cases (87.5%) according to the catheterization report. Supplementary Table S4 summarizes the baseline and clinical characteristics of each patient in the study cohort. Most procedures were elective/chronic presentations (29/32, 90.6%), while NSTEMI or unstable angina accounted for 3/32 cases (9.4%). Target vessels included the LAD in 11 cases (34.4%), LCx in 13 cases (40.6%), and RCA in eight cases (25.0%); no left main lesions were included. Moderate-to-severe calcification was present in 13 cases (40.6%), lesion length > 20 mm in six cases (18.8%), moderate-to-severe tortuosity in three cases (9.4%), and bifurcation lesions in 0 cases.

### 3.1. Agreement Between Predicted and Measured iFR

Predicted iFR values were averaged across angiographic images for each vessel. The No-RAG model demonstrated a mean absolute error (MAE) of 0.064 and RMSE of 0.083. The RAG model reduced MAE to 0.029 and RMSE to 0.038 (Table 1).

**Figure 1:**
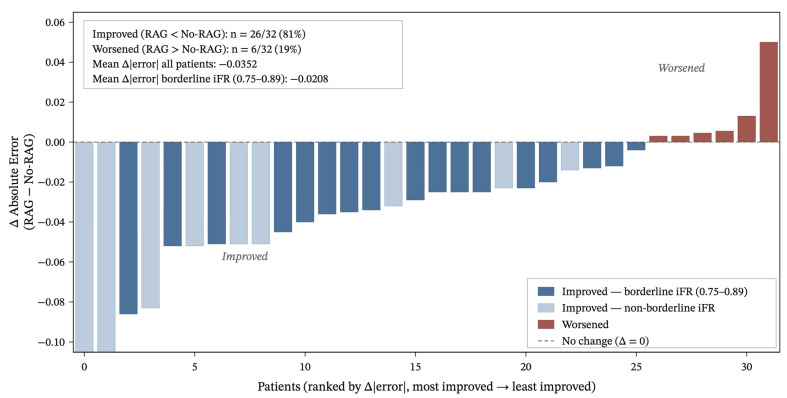
Case-Level Change in Absolute Prediction Error (RAG − No-RAG). Waterfall plot showing the case-level change in absolute prediction error between retrieval-augmented generation (RAG) and no retrieval augmentation (No-RAG), calculated as |error_RAG| − |error_No-RAG| for predicted instantaneous wave-free ratio (iFR). Negative values indicate lower absolute error with RAG and therefore improved agreement with invasive iFR, whereas positive values indicate higher absolute error with RAG.

Bootstrap resampling (15,000 iterations) demonstrated a statistically significant reduction in absolute error, with ΔMAE (RAG − No-RAG) 95% CI −0.052 to −0.021. Waterfall analysis demonstrated that most patients experienced a reduction in absolute prediction error with RAG, with only a minority showing minimal change or an increase in error (Figure 1). In a stratified paired bootstrap sensitivity analysis that preserved the original 25/7 ischemia-positive/negative distribution, the direction of the findings remained unchanged. RAG remained associated with lower prediction error than No-RAG, with ΔMAE of −0.035 and a stratified bootstrap 95% CI of −0.056 to −0.018. AUC also remained higher with RAG than No-RAG: 0.931 vs. 0.583, with a stratified bootstrap ΔAUC of +0.349 and 95% CI +0.043 to +0.652. These intervals remain wide and should be interpreted as exploratory, given the small number of non-ischemic lesions.

**Figure 2:**
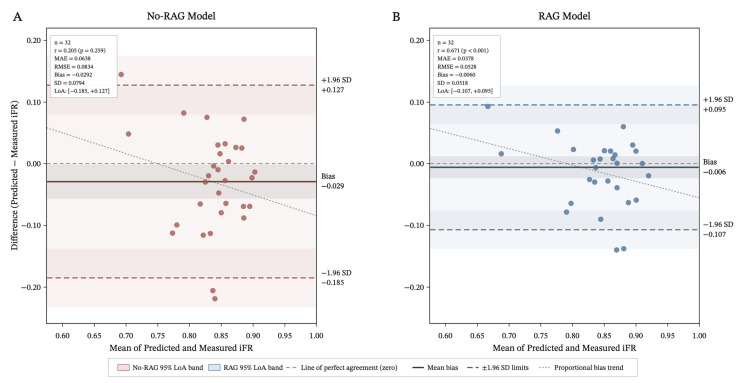
Bland–Altman Analysis of Predicted vs. Measured iFR. Bland–Altman plots comparing model-predicted and invasively measured instantaneous wave-free ratio (iFR) for the (**A**) No-RAG and (**B**) RAG conditions. The solid horizontal line represents mean bias (predicted minus measured iFR), and the dashed lines represent the 95% limits of agreement.

Bland–Altman analysis showed a bias of −0.029 for No-RAG (95% limits of agreement: −0.185 to +0.127) and −0.005 for RAG (−0.080 to +0.071), indicating reduced dispersion and improved agreement with retrieval augmentation (Figure 2).

**Figure 3:**
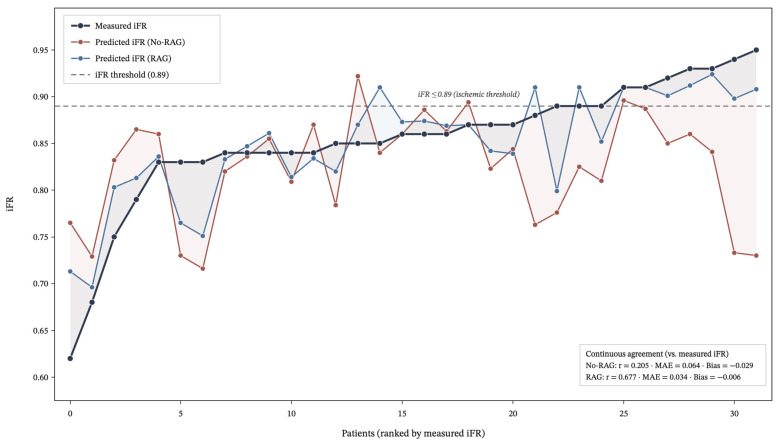
Correlation Between Predicted and Invasive iFR. Line plot showing measured invasive iFR and model-predicted iFR values for the No-RAG and RAG conditions across all cases, with patients ranked in ascending order by measured invasive iFR. The dashed horizontal line indicates the prespecified ischemic threshold of iFR ≤ 0.89.

Pearson correlation with invasive iFR increased from r = 0.205 (*p* = 0.259) for No-RAG to r = 0.830 (*p* < 0.001) for RAG, representing a strong association under RAG, compared with a weak association under No-RAG (Figure 3). The between-model difference in correlation reached statistical significance (Δr = +0.625, *p* < 0.001, using Fisher’s z-transformation). However, this should be interpreted only as a stronger linear association between predicted and measured iFR, not as evidence of agreement.

Additional calibration metrics were directionally consistent with the primary error-based analyses. Calibration-in-the-large improved from −0.029 with No-RAG to −0.005 with RAG, and Lin’s concordance correlation coefficient increased with RAG, supporting improved agreement beyond correlation alone. Exploratory hard-label Brier score also improved, consistent with the higher classification accuracy observed under RAG (Supplementary Table S5).

### 3.2. Model Performance for iFR-Defined Ischemia 

Using invasive iFR ≤ 0.89 as the reference standard, the cohort was markedly imbalanced, with 25 ischemia-positive lesions and only seven non-ischemic lesions. Accordingly, prevalence-dependent metrics such as accuracy and PPV should be interpreted cautiously. For context, a classifier that assigns all lesions as ischemic would achieve an apparent accuracy of 25/32 (78.1%) in this cohort.

The No-RAG model demonstrated sensitivity 0.920 (95% CI 0.75–0.98), specificity 0.143 (95% CI 0.03–0.51), accuracy 0.750 (95% CI 0.58–0.87), and AUC 0.583 (bootstrap 95% CI 0.30–0.85) (Table 2). Confusion matrices are shown in Table 2.

Precision–recall curves were also included as a supplementary analysis, using predicted iFR as the continuous ischemia score, with lower predicted iFR indicating greater likelihood of ischemia (Supplementary Figure S1). The baseline precision was 0.781, reflecting a high prevalence of ischemia. The area under the precision–recall curve was 0.82 for No-RAG and 0.98 for RAG. These analyses are descriptive only, given the small sample size and high class imbalance.

In the RAG condition, no false-positive ischemia classifications occurred among the seven non-ischemic lesions, yielding a specificity point estimate of 1.00. In the RAG model, sensitivity was 0.880 (95% CI 0.70–0.96); specificity increased substantially to 1.000 (95% CI 0.65–1.00); accuracy increased to 0.906 (95% CI 0.76–0.97); and AUC increased to 0.931 (bootstrap 95% CI 0.83–1.00). The difference in AUC (ΔAUC = +0.349) was statistically significant (*p* = 0.010; bootstrap 95% CI +0.081 to +0.628). However, this estimate was based on a very small denominator and is highly unstable; a single false-positive classification would have reduced specificity to 0.86. Therefore, specificity should be interpreted descriptively and not as robust evidence of diagnostic performance.

As a supplemental sensitivity analysis, translating predicted iFR into a binary PCI recommendation yielded better agreement with operator management under RAG compared to no RAG (accuracy 0.781 vs. 0.969; McNemar *p* = 0.032), although this analysis was based on only four operator no-PCI cases (Supplementary Table S6). Because the cohort was enriched for physiologically significant lesions, with 25 ischemia-positive cases and only seven ischemia-negative cases, threshold-based performance metrics should be interpreted cautiously. In particular, the RAG specificity of 1.00 was derived from correct classification of only seven non-ischemic lesions. This estimate is therefore highly unstable and should be viewed as a descriptive point estimate rather than evidence of robust diagnostic specificity.

### 3.3. Lesion Description and Interpretive Framing Shifts

In paired categorical comparisons, RAG reduced CABG mention (65.6% vs. 40.6%, *p* = 0.031; Table 3). Type C and Type B2 lesion labels also became less common, although these changes were not statistically significant. In contrast, wording that IVUS was “strongly favored” became more common with RAG (59.4% vs. 84.4%, *p* = 0.039). Overall, these shifts are consistent with a change in how the model framed lesion severity and physiologic uncertainty, and may partly explain the improved iFR prediction. These differences coincided with better performance but do not indicate improved reasoning or a causal link to iFR prediction.

### 3.4. Qualitative Mechanistic Analysis

To explore potential mechanisms by which retrieval may have improved iFR calibration, we conducted an exploratory structured qualitative review of paired model outputs (Table 4 and Supplementary Table S7).

In No-RAG outputs, the model usually centered its interpretation on angiographic appearance. Lesions were commonly described as “long diffuse disease,” “focal high-grade stenosis,” or “branch involvement,” and the narrative often moved quickly to action-oriented language such as “elective PCI” or “provisional bifurcation strategy.” Clinical details were often listed, but they were not consistently used to explain physiologic significance. Uncertainty, when present, usually appeared late and had little effect on the implied estimate of lesion severity.

With RAG, the same angiographic findings were more often placed within an explicit physiology-guided framework. The outputs more frequently referred to physiologic assessment, decision thresholds, and supporting trial or guideline language before committing to an estimate of lesion significance. Clinical syndrome framing, such as “chronic coronary syndrome” or “stable ischemic heart disease,” was also introduced earlier.

This difference was most apparent in borderline lesions. RAG outputs more often expressed graded uncertainty and emphasized physiologic confirmation before moving to procedural planning, whereas No-RAG outputs more often moved directly from morphology to intervention-oriented language. RAG outputs also more consistently mentioned post-procedural physiologic targets, suggesting that retrieval changed the model’s framing of lesion severity rather than simply making it more aggressive or less aggressive.

These descriptive differences were observed alongside improved quantitative performance but should not be interpreted as evidence of better reasoning or as identifying a causal mechanism for improved iFR prediction.

To assess whether selecting the lowest technically adequate invasive iFR biased the findings toward ischemic classification, we repeated the continuous agreement analysis using the mean of all technically adequate iFR measurements when multiple values were available. Multiple technically adequate iFR measurements were available in 9/32 vessels (28.1%). Using this alternative reference standard, the direction of the findings was unchanged. RAG continued to show lower prediction error than No-RAG, with MAE 0.032 versus 0.067 and RMSE 0.042 versus 0.086, respectively. The between-model difference in MAE remained favorable for RAG: ΔMAE −0.035, bootstrap 95% CI −0.052 to −0.018. Bland–Altman bias also remained closer to zero with RAG than with No-RAG: −0.011 versus −0.035. These findings suggest that the observed improvement with RAG was not driven solely by selecting the lowest iFR value as the primary reference standard. However, this finding remains hypothesis-generating given the sample size.

## 4. Discussion

In this retrospective, single-center pilot study of 32 vessels undergoing invasive iFR assessment, we evaluated whether retrieval augmentation changed LLM-generated iFR estimates and their alignment with clinically measured invasive iFR. Within this selected cohort, several key findings emerged: (1) continuous agreement with invasive iFR improved, with reduced absolute error (MAE 0.064 to 0.029) and stronger correlation (r = 0.205 to 0.830, *p* < 0.001), accompanied by narrower limits of agreement (±0.156 to ±0.075); (2) discrimination for ischemia (iFR ≤ 0.89) also improved (AUC 0.58 to 0.93, *p* = 0.010); (3) qualitative analysis demonstrated a systematic shift from morphology-dominant procedural framing toward earlier integration of physiologic thresholds, guideline references, and graded uncertainty. Given the limited sample size, these findings should not be interpreted as evidence that RAG reliably improves clinical decision-making. Rather, they suggest that RAG may influence the structure and emphasis of the model’s output in a way that is more consistent with physiology-guided coronary assessment. Likewise, although RAG reduced false-positive ischemia classifications in this cohort, the specificity estimate should not be overinterpreted. The value of 1.00 was derived from correct classification of only seven non-ischemic lesions, making it highly sensitive to even one additional false-positive result. Thus, the finding is best viewed as hypothesis-generating evidence that RAG may reduce angiography-driven overcalling of ischemia, rather than as proof of reliable specificity.

These findings should not be interpreted as the model “looking up” the correct iFR. The retrieved documents did not contain lesion-specific information or new visual detail about the angiogram. Notably, among lesions with measured iFR > 0.89, both the No-RAG and RAG models still often assigned values within or near the ischemic range, indicating a persistent tendency to overcall physiologic significance based on angiographic appearance alone. A more plausible explanation for the improvement with RAG is therefore indirect recalibration: the retrieved text may have helped the model apply physiologic thresholds more consistently, handle borderline lesions more cautiously, and reduce anatomy-only overcalling, even though reliable exclusion of ischemia remained limited. That interpretation is consistent with the observed reduction in false-positive ischemia calls, but it remains hypothesis-generating. Rather than demonstrating that RAG can reliably predict coronary physiology in clinical practice, these results suggest that retrieval augmentation may improve the internal alignment of LLM-generated estimates with invasive iFR measurements under a controlled retrospective workflow. This pattern is visually more apparent in Figure 3. Because Figure 3 displays predicted and measured iFR values across the measured iFR range, it shows that much of the visible disparity between No-RAG and RAG occurred among lesions with less severe, higher measured iFR values. In this range, No-RAG tended to assign predicted values closer to the ischemic threshold, suggesting overestimation of physiologic severity, whereas RAG appeared to scale these estimates more conservatively.

This distinction matters because angiographic appearance alone is an imperfect surrogate for lesion-specific ischemia. In routine practice, physiology-guided assessment is especially valuable for angiographically intermediate or borderline lesions, where anatomy alone may not reflect functional significance. In that setting, RAG may help constrain how the model translates angiographic appearance into an estimated physiologic severity by reinforcing established thresholds, deferral logic, and more explicit handling of uncertainty. Moreover, because this approach relies on routine angiographic images and text-based retrieval rather than pressure-wire instrumentation or dedicated computational physiology software [12], it may be particularly attractive for lower-resource settings if externally validated. However, its current role remains hypothesis-generating. The cohort was small and enriched for physiologically significant disease.

The magnitude of the observed improvement with RAG should be interpreted cautiously. The increase in linear association, reduction in MAE, and apparent increase in AUC are larger than would be expected from adding general guideline text to two static angiographic frames, particularly because the retrieved documents did not contain lesion-specific measurements, cine information, vessel reconstruction, or patient-specific flow modeling. This is clinically and biologically surprising: coronary physiology depends on pressure loss across the full interrogated vessel, lesion length, serial disease, diffuse disease, vessel caliber, subtended myocardial bed, microvascular conditions, and contrast-flow dynamics, many of which are incompletely represented by two still frames. This distinction is important when comparing the present exploratory LLM workflow with dedicated angiography-derived physiology tools. Angiography-derived FFR approaches have generally relied on quantitative coronary angiography, vessel segmentation/reconstruction, and computational or mathematical pressure-drop modeling. In a meta-analysis of 13 studies comprising 1842 vessels, angiography-derived FFR showed pooled sensitivity of 89%, specificity of 90%, and summary AUC of 0.84 versus invasive FFR [13]. Dedicated QFR and μQFR approaches have also been evaluated in substantially larger datasets; for example, μQFR was computed in 330 vessels from 306 patients and showed r = 0.90, mean difference 0.00 ± 0.05, and vessel-level diagnostic accuracy of 93.0% versus FFR [14]. Similarly, FAST II reported vFFR accuracy, sensitivity, and specificity of 90%, 81%, and 95%, respectively, while FAST-FFR reported FFRangio diagnostic accuracy of 92% [15,16]. The present LLM approach differs fundamentally from these systems because it did not perform validated three-dimensional reconstruction, contour extraction, computational fluid dynamics, or pressure-drop modeling. Accordingly, the present findings should not be interpreted as demonstrating that RAG enables reliable physiologic prediction from angiographic anatomy. A more conservative interpretation is that retrieval augmentation changed the model’s numeric calibration and narrative framing relative to the invasive iFR reference in this small, highly selected retrospective cohort. Potential explanations include threshold anchoring to guideline-derived iFR/FFR decision concepts, reduced morphology-driven overcalling of ischemia, regression toward the observed iFR range, chance performance variation in a small sample, and sensitivity of LLM outputs to prompt and retrieval context. These mechanisms could improve apparent alignment with invasive iFR without proving that the model has learned or extracted lesion-specific coronary physiology. Moreover, the model received only two orthogonal end-diastolic still frames and lacked access to cine angiography, vessel reconstruction, contrast-flow dynamics, computational fluid dynamics, pressure-wire pullback data, or other lesion-specific physiologic measurements. Therefore, the observed improvement should not be interpreted as demonstrating that RAG can reliably infer coronary physiology from anatomy.

The study population should also be interpreted as an iFR-selected lesion cohort rather than a routine coronary angiography population. In everyday practice, the decision to perform iFR is not random and may depend on angiographic ambiguity, lesion location, perceived procedural benefit, clinical presentation, operator judgment, and local workflow. This selection pathway may enrich the dataset with lesions near physiologic or treatment-decision thresholds and may reduce the representation of the full spectrum of coronary anatomy seen during diagnostic angiography. As a result, model performance in this cohort may not reflect performance in unselected angiographic populations that include a broader mix of normal vessels, mild disease, clearly severe stenoses, diffuse disease, complex multivessel disease, and lesions not considered suitable or necessary for invasive physiology. Similarly, because 25/32 lesions were ischemic, the cohort had a much higher prevalence of physiologically significant disease than would be expected in many routine diagnostic angiography populations. This imbalance may inflate prevalence-dependent metrics such as accuracy and PPV, while making specificity and NPV unstable because they are based on only seven non-ischemic lesions. Therefore, threshold-based classification results should be interpreted with caution and require validation in broader cohorts with lower, more representative ischemia prevalence. In a broader angiography cohort with lower ischemia prevalence, the apparent specificity gain observed with RAG may not be preserved. The current RAG specificity of 1.00 reflects 7/7 correctly classified non-ischemic lesions, and even one additional false-positive classification would reduce specificity to 0.86. A lower-prevalence cohort would include more non-ischemic lesions and a broader spectrum of mild, ambiguous, diffuse, and clinically non-actionable disease, which could reveal false-positive behavior not captured in this enriched sample. Such a cohort would also alter prevalence-dependent metrics, likely reducing PPV and changing apparent accuracy. Therefore, the observed specificity gain should be considered an unstable exploratory point estimate requiring validation in larger, lower-prevalence, multicenter cohorts. The present findings are better viewed as an early signal that retrieval augmentation may alter model calibration and framing than as evidence of reproducible performance gains.

### Study Limitations

Several limitations deserve emphasis. This was a retrospective single-center, single-operator pilot study with a small sample size. The cohort was selected from patients who underwent invasive physiology and, therefore, may not reflect broader angiography populations. The high prevalence of ischemic lesions limits generalizability and makes some performance estimates particularly sensitive to small changes in classification. Therefore, classification metrics, especially specificity and negative predictive performance, should be interpreted as exploratory and hypothesis-generating rather than definitive evidence of diagnostic accuracy. Although model versioning and inference settings are reported, LLM outputs may vary across model snapshots, API updates, inference settings, and repeated stochastic runs. Because repeat-run reproducibility was not formally tested, the observed results should be interpreted as the performance of this specific prompt, retrieval pipeline, and model configuration rather than as a deterministic property of GPT-5.2. Likewise, model outputs may vary with temperature, sampling settings, random seed control, model version, and repeated runs. Because no random seed or deterministic decoding mode was used and repeat-run reproducibility was not formally assessed, the results should be interpreted as the performance of this specific single-run inference configuration rather than as fully deterministic model behavior.

Use of two still frames rather than full angiographic cine runs is also a major limitation. Coronary physiologic significance depends on dynamic and spatial features that are incompletely captured by static images, including lesion length, serial disease, diffuse pressure loss, vessel tortuosity, contrast filling, distal vessel caliber, and myocardial bed subtended. Because GPT5.2 does not allow for cine angiography, vessel reconstruction, flow modeling, computational fluid dynamics, or pressure-wire pullback data, the results should be interpreted as exploratory model-output alignment with invasive iFR rather than validated image-based physiologic prediction.

It is also important to note that selecting the lowest technically adequate iFR may bias the reference standard toward ischemic classification, particularly in vessels with serial or diffuse disease. Because pullback data were not uniformly available, formal lesion-level adjudication of focal versus diffuse pressure loss was not possible. Therefore, the reference standard should be interpreted as distal vessel-level iFR rather than a lesion-specific physiologic map. This may be particularly relevant for diffuse disease, serial lesions, or complex lesion morphology. Moreover, decision-curve analysis was not performed because the model did not generate calibrated probabilities or prespecified treatment-utility estimates. Future studies with larger cohorts should evaluate decision-curve analysis using calibrated probabilistic outputs to determine whether model estimates provide net clinical benefit across clinically relevant decision thresholds.

The RAG condition depended on a specific document set, chunking strategy, embedding approach, and retrieval configuration, so the observed behavior may not generalize to other pipelines or source corpora. Because the retrieved corpus bundled clinical guidelines with a scientific statement on physiology, the present study cannot determine which, if any, of the retrieved content drove the observed changes. In particular, we cannot conclude that guideline statements alone improve image-based iFR prediction; they may have influenced thresholding, uncertainty handling, or interpretive framing rather than image interpretation itself. The RAG results may also depend on retrieval hyperparameters, including chunk size, overlap, similarity metric, and the number of retrieved chunks. Because we did not perform sensitivity analyses across alternative chunk sizes or k values, the observed performance should be interpreted as specific to this retrieval configuration rather than as evidence that the selected parameters are optimal. In addition, the study examined model outputs in a controlled retrospective setting and did not test whether these outputs improve clinician performance or can be safely integrated into real-world workflow.

Importantly, because no prespecified anatomic lesion-level exclusions were applied, the cohort may include features such as prior CABG anatomy, diffuse disease, ostial disease, heavy calcification, or suboptimal opacification, each of which can affect angiographic interpretation. This heterogeneity reflects the real-world iFR-selected cohort but also limits precision and external validity in this small pilot study. Likewise, use of two still frames rather than full angiographic cine runs is a major limitation. Coronary physiologic significance may depend on dynamic and spatial features better assessed on cine, including lesion length, serial lesions, vessel tortuosity, contrast filling, distal vessel size, and myocardial bed subtended. As a result, the model’s image input was incomplete relative to clinical angiographic interpretation, and the findings should not be assumed to reflect performance using full angiographic runs.

Because operator PCI decisions likely incorporated factors beyond iFR, including anatomy, symptoms, clinical presentation, lesion complexity, procedural feasibility, and intraprocedural judgment, the exploratory comparison between model-derived PCI recommendations and operator management should not be interpreted as validation against a ground-truth endpoint, but only as a descriptive assessment of concordance with observed clinical practice. Importantly, this study did not include a blinded human interventional cardiologist comparator; therefore, we cannot determine whether the LLM or RAG workflow performs better, worse, or similarly to expert visual angiographic assessment, and future work should benchmark model outputs against experienced physicians reviewing the same cases.

Finally, potential failure modes include incomplete still-frame representation of coronary anatomy, absence of cine input, poor contrast opacification, vessel overlap or foreshortening, heavy calcification, ostial or left main lesions, CTOs, serial or diffuse disease, prior CABG anatomy, ambiguous target-vessel territory, and small distal myocardial bed size. Additional LLM-specific failure modes include prompt sensitivity, model-version drift, stochastic output variability, retrieval of irrelevant or overly general text, unsupported reasoning, and overconfident numeric iFR estimates despite limited visual information. These limitations support treating the present findings as hypothesis-generating and not deployment-ready.

## 5. Conclusions

Overall, this pilot study suggests that RAG may improve alignment between LLM-generated iFR estimates and invasively measured iFR in a small, highly selected retrospective cohort. These findings remain hypothesis-generating and require prospective multicenter validation before any conclusions can be drawn about predictive validity or clinical use. The retrieved material should not be viewed as directly supplying the correct iFR for an angiogram; rather, any benefit likely arises from indirect constraint of model reasoning, decision thresholds, and uncertainty handling. At the same time, the present results are too limited to support clinical use or strong performance claims. The qualitative analysis identified descriptive differences in output framing and terminology, but it was subjective and does not establish a causal mechanism for the quantitative findings. The observed differences may reflect prompt- or retrieval-induced changes in numeric calibration, threshold anchoring, or narrative structure rather than improved physiologic inference from angiographic images. The appropriate next step is larger validation in broader and less selected cohorts, with prespecified evaluation frameworks and careful attention to whether retrieval augmentation produces more reliable, more calibrated, and more clinically useful outputs.

## Figures and Tables

**Table 1:** Continuous Prediction Error, Agreement, and Association Metrics Between Predicted and Measured iFR.

Metric	No-RAG	RAG	Between-Model Difference	*p*-Value
Pearson r (vs. actual iFR)	0.205 (95% CI −0.157 to 0.518) (*p* = 0.259)	0.830 (95% CI 0.677 to 0.914) (*p* < 0.001)	Δr = +0.625 (95% CI +0.356 to +0.833)	<0.001
Mean Absolute Error (MAE)	0.064 (95% CI 0.047 to 0.083)	0.029 (95% CI 0.021 to 0.040)	ΔMAE −0.035 95% CI −0.052 to −0.021	<0.001 †
Root Mean Square Error (RMSE)	0.083 (95% CI 0.062 to 0.108)	0.038 (95% CI 0.028 to 0.051)	ΔRMSE −0.045 95% CI −0.075 to −0.021	0.002
Bias (Pred − Actual)	−0.029 (95% CI −0.058 to 0.000)	−0.005 (95% CI −0.019 to +0.009)	Δbias +0.024 95% CI −0.001 to +0.050	−0.061
95% Limits of Agreement	−0.185 to +0.127	−0.080 to +0.071	Narrowed with RAG	—
Limits-of-agreement width	0.312 95% CI 0.250 to 0.415	0.151 95% CI 0.121 to 0.201	Δwidth −0.161 95% CI −0.242 to −0.079	0.004

Abbreviations: RAG = retrieval-augmented generation. † Bootstrap 15,000 resamples; ΔMAE 95% CI −0.052 to −0.021.

**Table 2:** Ischemia Classification Performance (iFR ≤ 0.89).

Metric	No-RAG	RAG	Δ (RAG − No-RAG)	*p*-Value
Sensitivity	0.920 (95% CI 0.74–0.99)	0.880 (95% CI 0.70–0.96)	−0.040	—
Specificity	0.143 (95% CI 0.00–0.58)	1.00 (95% CI 0.65–1.00)	+0.857	—
Accuracy	0.750 (95% CI 0.57–0.89)	0.906 (95% CI 0.76–0.97)	+0.156	—
AUC	0.583 (bootstrap 95% CI 0.300–0.850)	0.931 (bootstrap 95% CI 0.83–1.00)	+0.349	0.010 †
Classification performance with confusion matrices and imbalance-aware metrics				
Metric	No-RAG	RAG		
True Positives	23	22		
False Negatives	2	3		
True Negatives	1	7		
False Positives	6	0		
Sensitivity	0.920	0.880		
Specificity	0.143	1.000		
PPV	0.793	1.000		
NPV	0.333	0.700		
Accuracy	0.750	0.906		
Balanced Accuracy	0.532	0.940		
AUPRC	0.820	0.982		

Abbreviations: AUC = area under curve; CI = confidence interval; iFR = instantaneous wave-free ratio; RAG = retrieval-augmented generation. † Bootstrap ΔAUC 95% CI 0.081 to 0.628. Note: *n* = 32 (25 positive/7 negative). Predicted positive is defined as predicted iFR ≤ 0.89.

**Table 3:** Lesion Description and Interpretive Framing Features.

Descriptor	No-RAG % (*n*)	RAG % (*n*)	McNemar *p*-Value
CABG mention	65.6% (21/32)	40.6% (13/32)	0.031
Type C lesion classification	87.5% (28/32)	68.8% (22/32)	0.109
Type B2 lesion classification	100% (32/32)	90.6% (29/32)	0.250
“Long diffuse” phrasing	81.3% (26/32)	62.5% (20/32)	0.125
“Focal high-grade stenosis” phrasing	75.0% (24/32)	53.1% (17/32)	0.070
Branch involvement (diagonal mention)	68.8% (22/32)	56.3% (18/32)	0.289
“Complex LAD disease” phrasing	71.9% (23/32)	50.0% (16/32)	0.078
Provisional bifurcation strategy mention	93.8% (30/32)	90.6% (29/32)	1.000
IVUS “strongly favored” phrasing	59.4% (19/32)	84.4% (27/32)	0.039

Abbreviations: CABG = coronary artery bypass graft; IVUS = intravascular ultrasound; LAD = left anterior descending artery; RAG = retrieval augmented generation.

**Table 4:** Exploratory Qualitative Comparison of No-RAG and RAG Output Patterns.

Domain	No-RAG Output Pattern (Verbatim Terminology)	RAG Output Pattern (Verbatim Terminology)	Descriptive Interpretation
A. Borderline Lesions and Threshold Behavior	Emphasis on: “long diffuse atherosclerotic disease in the LAD,” “at least one focal high-grade stenosis,” “branch involvement (diagonal…),” “elective PCI,” “provisional bifurcation PCI strategy,” “IVUS imaging strongly favored” Rapid transition to: “Recommended intervention strategy (elective PCI),” Uncertainty embedded late: “Uncertainty flag: … cannot state whether PCI is definitively indicated vs. optimal medical therapy or CABG consideration.”	Emphasis on: “Integrated diagnosis (angiogram + clinical context),” “Chronic coronary syndrome (stable ischemic heart disease),” “Physiology-guided assessment,” “Post-PCI iFR ≥ 0.95,” “Invasive epicardial coronary physiologic assessment,” Explicit citation of: “FAME trial,” “ILUMIEN III,” “ACC/AHA/SCAI Guideline for Coronary Artery Revascularization,” Interpretive shift: Uncertainty and physiology discussion appear earlier in reasoning	No-RAG reasoning is morphology-dominant and escalates rapidly to PCI planning. RAG integrates physiology and guideline framing before procedural escalation. Borderline cases show earlier acknowledgment of physiologic uncertainty.
B. Angiographic Severity Framing	Emphasis on: “Long diffuse disease,” “Focal high-grade stenosis,” “Complex LAD disease,” “Bifurcation strategy,” “IVUS strongly favored,” Procedural sequencing: “Pre-PCI planning” → “Provisional bifurcation PCI strategy” → “Post-PCI optimization”	Emphasis on: “Physiology guidance for intermediate lesions,” “Physiology-guided PCI vs. angiography,” “Post-PCI iFR targets,” Guideline references (FAME, ILUMIEN III, ACC/AHA/SCAI) Interpretive shift: “Angiographic findings” → “Physiologic context considered” → “Guideline-based pathway”	No-RAG prioritizes anatomic descriptors leading directly to procedural planning. RAG reframes angiography within a physiology-guided framework before recommending intervention.
C. Clinical Context Integration	Lists comorbidities descriptively: “Hypertension,” “Hyperlipidemia,” “Obesity (BMI 31),” “On DAPT (ASA + ticagrelor),” “ARB + spironolactone” Often not directly linked to physiologic reasoning	Clinical context embedded within diagnostic framing: “Chronic coronary syndrome,” “Stable ischemic heart disease,” Medication and renal considerations integrated: “contrast-associated AKI,” “baseline risk,” “monitor K/Cr” Linked to physiologic strategy: “Physiology-guided assessment,” “Post-PCI physiology can be considered”	No-RAG treats comorbidities as background descriptors. RAG integrates systemic context into decision-making and physiologic interpretation.
D. Elective vs. Clinical Syndrome Framing	Sequence commonly: “Angiographic disease” → “Recommended intervention strategy”	Sequence commonly: “Chronic coronary syndrome”/“Stable ischemic heart disease”/“Elective PCI setting” → “Angiographic findings” → “Physiology guidance” → “Guideline framework”	RAG embeds lesion interpretation within syndrome-based clinical framing before intervention planning. This sequencing suggests structured reasoning rather than direct morphology-driven escalation.

Abbreviations: ACC/AHA/SCAI = American College of Cardiology/American Heart Association/Society for Cardiovascular Angiography and Interventions; AKI = acute kidney injury; ASA = aspirin; Cr = creatinine; DAPT = dual antiplatelet therapy; FAME = Fractional Flow Reserve Versus Angiography for Multivessel Evaluation; iFR = instantaneous wave-free ratio; ILUMIEN III: OPTIMIZE PCI = OPtical Coherence Tomography (OCT) Compared to Intravascular Ultrasound (IVUS) and Angiography to Guide Coronary Stent Implantation: a Multicenter RandomIZEd Trial in Percutaneous Coronary Intervention (PCI); K = potassium; LAD = left anterior descending artery; PCI = percutaneous coronary intervention; RAG = retrieval augmented generation. Note: These exploratory qualitative and categorical output-pattern analyses were not adjusted for multiplicity; therefore, *p*-values should be interpreted as nominal and hypothesis-generating.

## Data Availability

The original contributions presented in this study are included in the article/Supplementary Material. Further inquiries can be directed to the corresponding author.

## Supplementary Materials

The following supporting information can be downloaded at: https://www.mdpi.com/article/10.3390/jcm15135253/s1, Supplementary Table S1. Prompt design for No-RAG and RAG LLM inference; Supplementary Table S2. TRIPOD LLM Checklist; Supplementary Table S3. Additional Data for Reproducibility. Supplementary Table S4. Cohort Data. Supplementary Table S5. Additional calibration and agreement metrics for predicted iFR. Supplementary Table S6. Sensitivity Analysis of PCI Recommendation Agreement With Operator Management. Supplementary Table S7. Structured Qualitative Comparison of Reasoning Components. Supplementary Figure S1. Precision–Recall Curves for Ischemia Classification.

## Abbreviations

ACS	Acute coronary syndromes
AUC	Area under the curve
CABG	Coronary artery bypass grafting
CI	Confidence interval
FAISS	Facebook AI Similarity Search
GPT	Generative Pretrained Transformer
iFR	Instantaneous wave-free ratio
IVUS	Intravascular ultrasound
LLM	Large language model
MAE	Mean absolute error
PCI	Percutaneous coronary intervention
RAG	Retrieval-augmented generation
RMSE	Root mean square error
