# Supplementary material for: Retrieval-Augmented Large Language Model for Angiographic Prediction of Coronary Physiology
Source: J Clin Med. 2026 Jul 5;15(13):5253. doi: 10.3390/jcm15135253 (PMC13363511; doi:10.3390/jcm15135253)
Supplement: Supplementary file 1 [file jcm-15-05253-s001.zip › jcm-4391216-supplementary.pdf]

Supplementary Table S1. Prompt design for No-RAG and RAG LLM inference

Prompt Component	Text/Details
System instruction	"You are a cardiology-focused large language model participating in a retrospective research study. You will be provided de-identified clinical information and 2 orthogonal coronary angiographic still frames from the same target vessel. Your task is to estimate the invasive instantaneous wave-free ratio (iFR) for the target vessel. Do not claim to know the measured iFR. Use only the provided case information and images. Provide a structured research output, not clinical advice. Explicitly acknowledge uncertainty when image quality, lesion complexity, diffuse disease, serial disease, vessel overlap, or distal bed size limits inference."
Shared case input template	"Case ID: [study ID]. Target vessel: [vessel if available]. Clinical information: demographics: [age/sex]; presentation/indication: [text]; medical history: [risk factors/comorbidities]; laboratory values: [available labs]; medications: [cardiac medications]. Angiographic input: Frame 1 and Frame 2 are orthogonal end-diastolic still frames at maximal vessel opacification from the target vessel."
No-RAG user prompt	"Review the provided clinical information and 2 angiographic still frames. Estimate the physiologic significance of the target-vessel lesion. Provide: 1) brief angiographic description; 2) predicted iFR for Frame 1; 3) predicted iFR for Frame 2; 4) averaged case-level predicted iFR; 5) ischemia classification using iFR ≤0.89 as ischemic and >0.89 as non-ischemic; 6) confidence level; 7) brief reasoning; and 8) key limitations of estimating iFR from still frames."
RAG-specific wrapper	"The following retrieved excerpts from coronary physiology and revascularization guidance are provided only as general context. Use them to calibrate interpretation of physiologic thresholds, uncertainty, and guideline-based framing. Do not treat the retrieved text as patient-specific information and do not assume

	it contains the measured iFR. Retrieved context: [top 5 retrieved chunks]. Now complete the same task using the same clinical information and angiographic frames.”
Output constraints	Required fields: `target_vessel`, `frame_1_predicted_iFR`, `frame_2_predicted_iFR`, `mean_predicted_iFR`, `ischemia_classification`, `confidence`, `angiographic_reasoning`, and `limitations`. Predicted iFR must be numeric, reported to 2 decimal places, and constrained to a clinically plausible range of 0.50–1.00. The model must provide a value even when uncertain, but must state uncertainty in the reasoning field.
Threshold rule	“Classify predicted ischemia as present when mean predicted iFR ≤0.89 and absent when mean predicted iFR >0.89.”
Prompt refinement history	“The prompt was refined before final inference to standardize output formatting and ensure extraction of a numeric predicted iFR. Refinement was limited to formatting clarity and did not use measured iFR values, outcome labels, MAE, AUC, or other performance results. After the final prompt template was locked, the same template was applied to all included cases in both No-RAG and RAG conditions.”
RAG retrieval settings	Corpus: 2023 chronic coronary disease guideline, 2025 ACS guideline, 2021 revascularization guideline, and AHA scientific statement on invasive coronary physiology. Chunking: 500 characters with 100-character overlap. Embeddings: OpenAI embeddings. Vector database: FAISS. Retrieved chunks per query: k = 5.
Blinding/data leakage control	“The measured invasive iFR value, final ischemia label, and operator management decision were not included in the prompt. Retrieved documents contained general coronary physiology and revascularization guidance, not patient-specific lesion labels or measured iFR values.”

Model settings	Model: GPT version 5.2. Top-p: 0.98. Verbosity: medium. Temperature: 0.2. Runs per case: Single inference per condition per angiographic frame

Note: Because LLM outputs are sensitive to prompt wording and model version, this prompt table is provided to support reproducibility. Results should be interpreted in relation to this exact prompt, model configuration, and RAG retrieval pipeline.

Supplementary Table S2. TRIPOD LLM Checklist

Section	Item	Checklist Item	Research Design	LLM Task	Page
Title	1	Identify the study as developing, fine-tuning, and/or evaluating the performance of an LLM, specifying the task, the target population, and the outcome to be predicted.	All	All	MS p. 1
Abstract	2	See TRIPOD-LLM for Abstracts.	All	All	MS pp. 1-2
Introduction / Background	3a	Explain the healthcare context / use case (e.g., administrative, diagnostic, therapeutic, clinical workflow) and rationale for developing or evaluating the LLM, including references to existing approaches and models.	All	All	MS pp. 2-3
Introduction / Background	3b	Describe the target population and the intended use of the LLM in the context of the care pathway, including its intended users in current gold standard practices (e.g., healthcare professionals, patients, public, or administrators).	E/H	All	MS pp. 3, 6
Introduction / Objectives	4	Specify the study objectives, including whether the study describes the initial development, fine-tuning, or validation of an LLM (or multiple stages).	All	All	MS p. 3
Methods / Data	5a	Describe the sources of data separately for the training, tuning, and/or evaluation datasets and the rationale for using these data (e.g., web corpora, clinical research/trial data, EHR data, or unknown).	All	All	MS pp. 3-4, 6
Methods / Data	5b	Describe the relevant data points and provide a quantitative and qualitative description of their distribution and other relevant descriptors of the dataset (e.g., source, languages, countries of origin).	All	All	MS pp. 9-10; Supplementary Table 4
Methods / Data	5c	Specifically state the date of the oldest and newest item of text used in the development process (training, fine-tuning, reward modeling) and in the evaluation datasets.	All	All	MS pp. 3, 6
Methods / Data	5d	Describe any data pre-processing and quality checking, including whether this was similar across text corpora, institutions, and relevant socio-demographic groups.	All	All	MS pp. 4, 6-7
Methods / Data	5e	Describe how missing and imbalanced data were handled and provide reasons for omitting any data.	All	All	MS pp. 3-4, 9, 22-24
Methods / Analytical Methods	6a	Report the LLM name, version, and last date of training.	All	All	MS p. 5
Methods / Analytical Methods	6b	Report details of LLM development process, such as LLM architecture, training, fine-tuning procedures, and alignment strategy (e.g., reinforcement learning, direct preference optimization, etc.) and alignment goals (e.g., helpfulness, honesty, harmlessness, etc.).	M/D	All	N/A: existing closed-source LLM; no de novo development/fine-tuning performed (MS p. 5)
Methods / Analytical Methods	6c	Report details of how text was generated using the LLM, including any prompt engineering (including consistency of outputs), and inference settings (e.g., seed, temperature, max token length, penalties), as relevant.	M/D/E	All	MS p. 5; Supplementary Table 1

Section	Item	Checklist Item	Research Design	LLM Task	Page
Methods / Analytical Methods	6d	Specify the initial and post-processed output of the LLM (e.g., probabilities, classification, unstructured text).	All	All	MS pp. 5, 8
Methods / Analytical Methods	6e	Provide details and rationale for any classification and, if applicable, how the probabilities were determined and thresholds identified.	All	C/OF	MS pp. 5, 8, 14-15
Methods / LLM Output	7a	Include metrics that capture the quality of generative outputs, such as consistency, relevance, and accuracy, compared to gold standards.	All	QA/IR/DG/SS/ MT	MS pp. 8, 10-15; Tables 1-2
Methods / LLM Output	7b	Report the outcome metrics' relevance to downstream task at deployment time and, where applicable, correlation of metric to human evaluation of the text for the intended use.	E/H	All	MS pp. 8, 14-15, 22-24
Methods / LLM Output	7c	Clearly define the outcome, how the LLM predictions were calculated (e.g., formula, code, object, API), the date of inference for closed-source LLMs, and evaluation metrics.	E/H	All	MS pp. 5, 8-9, 10-15
Methods / LLM Output	7d	If outcome assessment requires subjective interpretation, describe the qualifications of the assessors, any instructions provided, relevant information on demographics of the assessors, and inter-assessor agreement.	All	All	MS pp. 7, 9
Methods / LLM Output	7e	Specify how performance was compared to other LLMs, humans, and other benchmarks or standards.	All	All	MS pp. 21, 24
Methods / Annotation	8a	If annotation was done, report how text was labeled, including providing specific annotation guidelines with examples.	All	All	MS pp. 7, 9; Table 4 pp. 17-18
Methods / Annotation	8b	If annotation was done, report how many annotators labeled the dataset(s), including the proportion of data in each dataset that were annotated by more than 1 annotator, and the inter-annotator agreement.	All	All	MS pp. 7, 9
Methods / Annotation	8c	If annotation was done, provide information on the background and experience of the annotators or characteristics of any models involved in labelling.	All	All	MS pp. 7, 9
Methods / Prompting	9a	If research involved prompting LLMs, provide details on the processes used during prompt design, curation, and selection.	All	All	MS p. 5; Supplementary Table 1
Methods / Prompting	9b	If research involved prompting LLMs, report what data were used to develop the prompts.	All	All	MS p. 5; Supplementary Table 1
Methods / Summarization	10	Describe any preprocessing of the data before summarization.	All	SS	N/A: no preprocessing or altering of data before summarization task
Methods / Instruction tuning/Alignment	11	If instruction tuning/alignment strategies were used, what were the instructions, data, and interface used for evaluation, and what were the characteristics of the populations doing evaluation?	M/D	All	N/A: no instruction tuning or alignment performed

Section	Item	Checklist Item	Research Design	LLM Task	Page
Methods / Compute	12	Report compute, or proxies thereof (e.g., time on what and how many machines, cost on what and how many machines, inference time, floating-point operations per second [FLOPs]), required to carry out methods.	M/D/E	All	MS p. 5 (environment/API); compute time/cost not reported
Methods / Ethical Approval	13	Name the institutional research board or ethics committee that approved the study and describe the participant-informed consent or the ethics committee waiver of informed consent.	All	All	MS pp. 5, 26
Methods / Open Science	14a	Give the source of funding and the role of the funders for the present study.	All	All	MS p. 25
Methods / Open Science	14b	Declare any conflicts of interest and financial disclosures for all authors.	All	All	MS p. 26
Methods / Open Science	14c	Indicate where the study protocol can be accessed or state that a protocol was not prepared.	H	All	Not reported in manuscript
Methods / Open Science	14d	Provide registration information for the study, including register name and registration number, or state that the study was not registered.	H	All	Not reported in manuscript
Methods / Open Science	14e	Provide details of the availability of the study data.	All	All	MS p. 26
Methods / Open Science	14f	Provide details of the availability of the code to reproduce the study results.	All	All	MS p. 25; Supplementary Appendix/statistical code
Methods / Public Involvement	15	Provide details of any patient and public involvement during the design, conduct, reporting, interpretation, or dissemination of the study or state no involvement.	H	All	Not reported in manuscript
Results / Participants	16a	When using patient/EHR data, describe the flow of text/EHR/patient data through the study, including the number of documents/questions/participants with and without the outcome/label and follow-up time as applicable.	E/H	All	MS pp. 9-10; Figure 1
Results / Participants	16b	When using patient/EHR data, report the characteristics overall and, for each data source or setting, and for development/evaluation splits, including the key dates, key characteristics, and sample size.	E/H	All	MS pp. 9-10; Supplementary Table 4
Results / Participants	16c	For LLM evaluation that include clinical outcomes, show a comparison of the distribution of important clinical variables that may be associated with the outcome between development and evaluation data, if available.	E/H	All	MS pp. 9-10; single evaluation cohort, no development/evaluation split
Results / Participants	16d	When using patient/EHR data, specify the number of participants and outcome events in each analysis (e.g., for LLM development, hyperparameter tuning, LLM evaluation).	E/H	All	MS pp. 9, 14-15
Results / Performance	17	Report LLM performance according to pre-specified metrics (see item 7a) and/or human evaluation (see item 7d).	All	All	MS pp. 10-18; Tables 1-4
Results / LLM Updating	18	If applicable, report the results from any LLM updating, including the updated LLM and subsequent performance.	All	All	N/A: no LLM updating performed; model snapshot caveat MS pp. 5, 23

Section	Item	Checklist Item	Research Design	LLM Task	Page
Discussion / Interpretation	19a	Give an overall interpretation of the main results, including issues of fairness in the context of the objectives and previous studies.	All	All	MS pp. 19-22
Discussion / Limitations	19b	Discuss any limitations of the study and their effects on any biases, statistical uncertainty, and generalizability.	All	All	MS pp. 22-24
Discussion / Usability of LLM in context	19c	Describe any known challenges in using data for the specified task and domain context with reference to representation, missingness, harmonization, and bias.	E/H	All	MS pp. 22-24
Discussion / Usability of LLM in context	19d	Define the intended use for the implementation under evaluation, including the intended input, end-user, level of autonomy/human oversight.	E/H	All	MS pp. 6, 24-25
Discussion / Usability of LLM in context	19e	If applicable, describe how poor quality or unavailable input data should be assessed and handled when implementing the LLM, i.e., what is the usability of the LLM in the context of current clinical care.	E/H	All	MS pp. 3-4, 23-24
Discussion / Usability of LLM in context	19f	If applicable, specify whether users will be required to interact in the handling of the input data or use of the LLM, and what level of expertise is required of users.	E/H	All	MS pp. 6, 24
Discussion / Usability of LLM in context	19g	Discuss any next steps for future research, with a specific view to applicability and generalizability of the LLM.	All	All	MS pp. 24-25

Abbreviations: LLM = large language model; M = LLM methods; D = de novo LLM development; E = LLM evaluation; H = LLM evaluation in healthcare settings; C = classification; OF = outcome forecasting; QA = long-form question-answering; IR = information retrieval; DG = document generation; SS = summarization and simplification; MT = machine translation.

Supplementary Table S3. Additional Data for Reproducibility

**Prompt Templates**	
**System Prompt**	SYSTEM PROMPT<br>You are a cardiology-focused large language model participating in a retrospective research study. You will receive de-identified structured clinical information and two orthogonal coronary angiographic still frames from the same target vessel. Your task is to estimate the invasive instantaneous wave-free ratio (iFR) of the target vessel for research purposes only.<br><br>Rules:<br>1. Do not claim to know the measured invasive iFR; it is not provided to you.<br>2. Use only the supplied clinical information, angiographic still frames, and, when applicable, retrieved reference text.<br>3. Do not provide clinical care instructions or autonomous treatment decisions.<br>4. Estimate a numeric iFR between 0.50 and 1.00, rounded to two decimal places, for each angiographic frame and provide a case-level mean predicted iFR.<br>5. Classify predicted ischemia using the prespecified threshold: iFR <= 0.89 = ischemic; iFR > 0.89 = non-ischemic.<br>6. Explicitly state uncertainty when lesion length, serial lesions, diffuse disease, ostial/left main anatomy, vessel overlap, foreshortening, poor opacification, heavy calcification, tortuosity, distal bed size, or still-frame limitations affect interpretation.<br>7. Keep the output structured and concise. Avoid overconfident statements.
**No-RAG user prompt**	SHARED CASE PROMPT TEMPLATE FOR NO-RAG CONDITION<br>Case ID: {case_id}<br>Target vessel: {target_vessel}<br>Clinical information:<br>- Demographics: {age}, {sex}<br>- Presentation/indication: {presentation_or_indication}<br>- Medical history/risk factors: {medical_history}<br>- Laboratory values: {laboratory_values}<br>- Medications: {medications}<br><br>Angiographic image input:<br>- Frame 1: Orthogonal end-diastolic still frame at maximal target-vessel opacification.

	- Frame 2: Orthogonal end-diastolic still frame at maximal target-vessel opacification.<br><br>Task:<br>Review the structured clinical information and the two angiographic still frames. Estimate the target-vessel iFR for each frame and provide the averaged case-level predicted iFR. Use the iFR threshold <=0.89 to classify predicted ischemia.<br><br>Required output fields:<br>1. case_id<br>2. target_vessel<br>3. angiographic_description<br>4. frame_1_predicted_iFR<br>5. frame_2_predicted_iFR<br>6. mean_predicted_iFR<br>7. ischemia_classification using iFR <=0.89<br>8. confidence level: low / moderate / high<br>9. key reasoning in 3-5 sentences<br>10. limitations of inference from still angiographic frames<br>11. optional exploratory PCI recommendation, clearly labeled as non-decisional research output
**RAG wrapper prompt**	The following retrieved excerpts are provided as general coronary physiology and revascularization context. They are not patient-specific and do not contain the measured iFR for this case. Use them only to calibrate threshold interpretation, physiologic uncertainty, and guideline-based framing. Do not treat retrieved text as a substitute for the angiographic images or clinical information.<br><br>Retrieved context:<br>{retrieved_chunk_1}<br>{retrieved_chunk_2}<br>{retrieved_chunk_3}<br>{retrieved_chunk_4}<br>{retrieved_chunk_5}<br><br>Now complete the same task using the structured case information and the two angiographic still frames.

	Use the same required output fields as in the No-RAG condition.
**Output schema**	STRUCTURED OUTPUT SCHEMA<br>{<br>"case_id": "{case_id}",<br>"target_vessel": "{target_vessel}",<br>"frame_1_predicted_iFR": 0.00,<br>"frame_2_predicted_iFR": 0.00,<br>"mean_predicted_iFR": 0.00,<br>"ischemia_classification": "ischemic or non-ischemic using iFR <=0.89",<br>"confidence": "low/moderate/high",<br>"angiographic_reasoning": "brief text",<br>"clinical_context_integration": "brief text",<br>"limitations": "brief text",<br>"exploratory_pci_recommendation": "not ground truth; non-decisional research output"
**Representative retrieved chunks**	
**Source**	**Retrieved Chunk Text**
AHA invasive physiology statement	Nonhyperemic pressure ratios such as iFR are used to assess physiologic significance of epicardial stenoses; iFR values at or below the accepted threshold support ischemia-producing disease.
2021 revascularization guideline	For angiographically intermediate stenoses, coronary physiology can guide PCI decisions, and physiology-positive lesions are more likely to benefit from revascularization than angiography alone would suggest.
AHA invasive physiology statement	Diffuse disease and serial lesions can cause cumulative pressure loss; distal pressure ratios may reflect combined vessel-level burden rather than a single focal stenosis.
2023 chronic coronary disease guideline	Management of stable coronary disease integrates symptoms, medical therapy, anatomy, ischemia burden, and patient context.
AHA invasive physiology statement	Post-PCI physiologic assessment can identify residual pressure gradients and may help evaluate procedural result, but interpretation depends on clinical context.
2021 revascularization guideline	When physiology is negative in stable intermediate lesions, deferral of PCI is generally supported; angiographic severity alone may overestimate functional significance.
AHA invasive physiology statement	Visual stenosis severity correlates imperfectly with physiologic significance, particularly in moderate lesions and complex anatomy.

2023 chronic coronary disease guideline	Guideline-directed medical therapy remains foundational in chronic coronary disease and revascularization decisions require clinical context.
**Statistical Analysis Code**	

```
# case_id, measured_ifr, no_rag_frame1, no_rag_frame2, rag_frame1, rag_frame2
# Optional columns: measured_ifr_mean, operator_pci, target_vessel, borderline_flag

# -----------------------------
# 1. Load data
# -----------------------------
infile <- "patient_level_ifr_predictions.csv"
df <- read.csv(infile, stringsAsFactors = FALSE)

required <- c("case_id", "measured_ifr", "no_rag_frame1", "no_rag_frame2",
              "rag_frame1", "rag_frame2")
missing_cols <- setdiff(required, names(df))
if (length(missing_cols) > 0) {
  stop("Missing required columns: ", paste(missing_cols, collapse = ", "))
}

row_mean2 <- function(a, b) rowMeans(cbind(a, b), na.rm = TRUE)

df$pred_no_rag <- row_mean2(df$no_rag_frame1, df$no_rag_frame2)
df$pred_rag    <- row_mean2(df$rag_frame1, df$rag_frame2)
df$actual_pos  <- df$measured_ifr <= 0.89

if (any(!is.finite(df$measured_ifr)) || any(!is.finite(df$pred_no_rag)) ||
    any(!is.finite(df$pred_rag))) {
  stop("Measured or predicted iFR contains missing/non-finite values after averaging.")
}

# -----------------------------
# 2. Metric functions
```

	```<br># -----------------------------<br>mae <- function(pred, obs) mean(abs(pred - obs))<br>rmse <- function(pred, obs) sqrt(mean((pred - obs)^2))<br>bias <- function(pred, obs) mean(pred - obs)<br>loa <- function(pred, obs) {<br>d <- pred - obs<br>c(lower = mean(d) - 1.96 * sd(d), upper = mean(d) + 1.96 * sd(d),<br>width = 3.92 * sd(d))<br>}<br>pearson_r <- function(pred, obs) unname(cor(pred, obs, method = "pearson"))<br><br>lins_ccc <- function(pred, obs) {<br>mx <- mean(pred); my <- mean(obs)<br>vx <- var(pred); vy <- var(obs)<br>covxy <- cov(pred, obs)<br>unname((2 * covxy) / (vx + vy + (mx - my)^2))<br>}<br><br>calibration_fit <- function(pred, obs) {<br>fit <- lm(obs ~ pred)<br>c(intercept = unname(coef(fit)[1]), slope = unname(coef(fit)[2]))<br>}<br><br>auc_rank <- function(actual_pos, pred_ifr) {<br># Lower iFR indicates higher ischemia probability, so use -pred_ifr as the score.<br>score <- -pred_ifr<br>y <- as.integer(actual_pos)<br>n1 <- sum(y == 1); n0 <- sum(y == 0)<br>if (n1 == 0 || n0 == 0) return(NA_real_)<br>r <- rank(score, ties.method = "average")<br>unname((sum(r[y == 1]) - n1 * (n1 + 1) / 2) / (n1 * n0))<br>}<br>```

	classification_metrics <- function(actual_pos, pred_ifr, threshold = 0.89) {<br>pred_pos <- pred_ifr <= threshold<br>tp <- sum(pred_pos & actual_pos)<br>tn <- sum(!pred_pos & !actual_pos)<br>fp <- sum(pred_pos & !actual_pos)<br>fn <- sum(!pred_pos & actual_pos)<br>sens <- ifelse(tp + fn > 0, tp / (tp + fn), NA_real_)<br>spec <- ifelse(tn + fp > 0, tn / (tn + fp), NA_real_)<br>ppv <- ifelse(tp + fp > 0, tp / (tp + fp), NA_real_)<br>npv <- ifelse(tn + fn > 0, tn / (tn + fn), NA_real_)<br>acc <- (tp + tn) / length(actual_pos)<br>brier_hard <- mean((as.integer(pred_pos) - as.integer(actual_pos))^2)<br>c(tp = tp, tn = tn, fp = fp, fn = fn, sensitivity = sens, specificity = spec,<br>ppv = ppv, npv = npv, accuracy = acc, brier_hard = brier_hard,<br>auc = auc_rank(actual_pos, pred_ifr))<br>}<br><br>exact_binom_ci <- function(x, n) {<br>if (n == 0) return(c(NA_real_, NA_real_))<br>unname(binom.test(x, n)$conf.int)<br>}<br><br>continuous_metrics <- function(dat) {<br>obs <- dat$measured_ifr<br>out <- rbind(<br>no_rag = c(mae = mae(dat$pred_no_rag, obs),<br>rmse = rmse(dat$pred_no_rag, obs),<br>bias = bias(dat$pred_no_rag, obs),<br>loa(dat$pred_no_rag, obs),<br>pearson = pearson_r(dat$pred_no_rag, obs),<br>ccc = lins_ccc(dat$pred_no_rag, obs),<br>calibration_fit(dat$pred_no_rag, obs)),<br>rag = c(mae = mae(dat$pred_rag, obs),

```
          rmse = rmse(dat$pred_rag, obs),
          bias = bias(dat$pred_rag, obs),
          loa(dat$pred_rag, obs),
          pearson = pearson_r(dat$pred_rag, obs),
          ccc = lins_ccc(dat$pred_rag, obs),
          calibration_fit(dat$pred_rag, obs))
  )
  as.data.frame(out)
}

# -----------------------------
# 3. Bootstrap uncertainty
# -----------------------------
boot_once <- function(dat, stratified = FALSE) {
  if (stratified) {
    pos_idx <- which(dat$actual_pos)
    neg_idx <- which(!dat$actual_pos)
    idx <- c(sample(pos_idx, length(pos_idx), replace = TRUE),
             sample(neg_idx, length(neg_idx), replace = TRUE))
  } else {
    idx <- sample(seq_len(nrow(dat)), nrow(dat), replace = TRUE)
  }
  bdat <- dat[idx, , drop = FALSE]
  obs <- bdat$measured_ifr
  c(
    no_rag_mae = mae(bdat$pred_no_rag, obs),
    rag_mae = mae(bdat$pred_rag, obs),
    delta_mae = mae(bdat$pred_rag, obs) - mae(bdat$pred_no_rag, obs),
    no_rag_rmse = rmse(bdat$pred_no_rag, obs),
    rag_rmse = rmse(bdat$pred_rag, obs),
    delta_rmse = rmse(bdat$pred_rag, obs) - rmse(bdat$pred_no_rag, obs),
    no_rag_bias = bias(bdat$pred_no_rag, obs),
    rag_bias = bias(bdat$pred_rag, obs),
```

	```<br>delta_bias = bias(bdat$pred_rag, obs) - bias(bdat$pred_no_rag, obs),<br>no_rag_auc = auc_rank(bdat$actual_pos, bdat$pred_no_rag),<br>rag_auc = auc_rank(bdat$actual_pos, bdat$pred_rag),<br>delta_auc = auc_rank(bdat$actual_pos, bdat$pred_rag) -<br>auc_rank(bdat$actual_pos, bdat$pred_no_rag),<br>no_rag_ccc = lins_ccc(bdat$pred_no_rag, obs),<br>rag_ccc = lins_ccc(bdat$pred_rag, obs),<br>delta_ccc = lins_ccc(bdat$pred_rag, obs) - lins_ccc(bdat$pred_no_rag, obs)<br>)<br>}<br><br>B <- 15000<br>boot_unstrat <- replicate(B, boot_once(df, stratified = FALSE))<br>boot_strat <- replicate(B, boot_once(df, stratified = TRUE))<br><br>ci <- function(x) quantile(x, probs = c(0.025, 0.975), na.rm = TRUE, names = FALSE)<br>boot_ci_unstrat <- t(apply(boot_unstrat, 1, ci))<br>boot_ci_strat <- t(apply(boot_strat, 1, ci))<br>colnames(boot_ci_unstrat) <- c("ci_lower", "ci_upper")<br>colnames(boot_ci_strat) <- c("ci_lower", "ci_upper")<br><br># -----------------------------<br># 4. Main outputs<br># -----------------------------<br>cont <- continuous_metrics(df)<br>class_no_rag <- classification_metrics(df$actual_pos, df$pred_no_rag)<br>class_rag <- classification_metrics(df$actual_pos, df$pred_rag)<br>class_table <- rbind(no_rag = class_no_rag, rag = class_rag)<br><br># Exact CIs for sensitivity, specificity, and accuracy<br>cis <- data.frame(<br>model = c("no_rag", "rag"),<br>sensitivity_low = c(exact_binom_ci(class_no_rag["tp"], class_no_rag["tp"] + class_no_rag["fn"])[1],<br>```

```
                    exact_binom_ci(class_rag["tp"], class_rag["tp"] + class_rag["fn"])[1]),
  sensitivity_high = c(exact_binom_ci(class_no_rag["tp"], class_no_rag["tp"] + class_no_rag["fn"])[2],
                     exact_binom_ci(class_rag["tp"], class_rag["tp"] + class_rag["fn"])[2]),
  specificity_low = c(exact_binom_ci(class_no_rag["tn"], class_no_rag["tn"] + class_no_rag["fp"])[1],
                    exact_binom_ci(class_rag["tn"], class_rag["tn"] + class_rag["fp"])[1]),
  specificity_high = c(exact_binom_ci(class_no_rag["tn"], class_no_rag["tn"] + class_no_rag["fp"])[2],
                     exact_binom_ci(class_rag["tn"], class_rag["tn"] + class_rag["fp"])[2]),
  accuracy_low = c(exact_binom_ci(class_no_rag["tp"] + class_no_rag["tn"], nrow(df))[1],
                 exact_binom_ci(class_rag["tp"] + class_rag["tn"], nrow(df))[1]),
  accuracy_high = c(exact_binom_ci(class_no_rag["tp"] + class_no_rag["tn"], nrow(df))[2],
                  exact_binom_ci(class_rag["tp"] + class_rag["tn"], nrow(df))[2])
)

# Paired exact McNemar-style comparison of correctness
no_correct <- (df$pred_no_rag <= 0.89) == df$actual_pos
rag_correct <- (df$pred_rag <= 0.89) == df$actual_pos
b <- sum(no_correct & !rag_correct)
c <- sum(!no_correct & rag_correct)
accuracy_mcnemar_p <- ifelse((b + c) > 0, binom.test(min(b, c), b + c, p = 0.5)$p.value, NA_real_)

# Optional sensitivity analysis using mean technically adequate invasive iFR
if ("measured_ifr_mean" %in% names(df)) {
  df_mean <- df
  df_mean$measured_ifr <- df_mean$measured_ifr_mean
  df_mean$actual_pos <- df_mean$measured_ifr <= 0.89
  cont_mean_reference <- continuous_metrics(df_mean)
  write.csv(cont_mean_reference, "continuous_metrics_mean_ifr_reference.csv")
}

# -----------------------------
# 5. Export results
# -----------------------------
write.csv(cont, "continuous_metrics_main.csv")
```

	```<br>write.csv(class_table, "classification_metrics_main.csv")<br>write.csv(cis, "classification_exact_cis.csv", row.names = FALSE)<br>write.csv(boot_ci_unstrat, "bootstrap_ci_unstratified.csv")<br>write.csv(boot_ci_strat, "bootstrap_ci_stratified_by_ifr_class.csv")<br>write.csv(data.frame(b = b, c = c, p_value = accuracy_mcnemar_p),<br>"paired_accuracy_mcnemar.csv", row.names = FALSE)<br><br>print(cont)<br>print(class_table)<br>print("Unstratified bootstrap CIs:")<br>print(boot_ci_unstrat)<br>print("Stratified bootstrap CIs:")<br>print(boot_ci_strat)<br>print(paste("Paired accuracy exact McNemar p =", signif(accuracy_mcnemar_p, 3)))<br>```

Supplementary Table S4. Cohort Data

	N = 32
**Clinical Characteristics**	
**Age**, years	65.7 ± 8.6
**Body mass index**, kg/m²	27.6 ± 6.1
**Female**	13 (40.6)
**Race**	
Caucasian	28 (87.5)
African American	2 (6.3)
Asian	2 (6.3)
**Hispanic Ethnicity**	4 (12.5)
**Comorbidities**	
Hypertension	30 (93.8)
Diabetes Mellitus	20 (62.5)
Hyperlipidemia	29 (90.6)
Atrial Fibrillation	3 (9.4)
Prior Myocardial Infarction	4 (12.5)
Prior PCI	11 (34.4)
Prior CABG	1 (3.1)
Prior Stroke	3 (9.4)
Peripheral Arterial Disease	3 (9.4)
Congestive Heart Failure	3 (9.4)
LVEF, %	55.4 ± 6.9
Estimated glomerular filtration rate	65.9 ± 30.1
Baseline Creatinine	1.57 ± 2.12
**Elective Procedure Status**	29 (90.6)
**NSTEMI or Unstable Angina**	3 (9.4)
**Medications**	
Statin	23 (71.9)
ACE Inhibitor/ARB	25 (78.1)

Beta-Blocker	21 (65.6)
Aspirin	23 (71.9)
P2Y12 inhibitor	10 (31.3)
**Angiographic Details**	
**Target Vessel**	
Left Main Artery	0 (0)
Left Anterior Descending Artery	11 (34.4)
Circumflex Artery	13 (40.6)
Right Coronary Artery	8 (25.0)
**Moderate to Severe Calcification**	13 (40.6)
**Bifurcation**	0 (0)
**Lesion Length > 20 mm**	6 (18.8)
**Moderate to Severe Tortuosity**	3 (9.4)

Abbreviations: ACE = angiotensin-converting enzyme; CABG = coronary artery bypass graft; NSTEMI = non-ST elevation myocardial infarction; PCI = percutaneous coronary intervention

Supplementary Table S5. Additional calibration and agreement metrics for predicted iFR

**Metric**	**No RAG**	**RAG**
Calibration-in-the-large / mean bias	−0.029	−0.005
Calibration slope	0.24	0.88
Lin's concordance correlation coefficient	0.18	0.79
Hard-label Brier score for ischemia classification	0.25	0.094

Abbreviations: RAG = retrieval-augmented generation

Supplementary Table S6. Sensitivity Analysis of PCI Recommendation Agreement With Operator Management

Metric	No-RAG	RAG	Δ (RAG − No-RAG)	p-value
Sensitivity	0.893 (95% CI 0.72–0.98)	1.00 (95% CI 0.88–1.00)	+0.107	—
Specificity	0.000 (95% CI 0.00–0.60)	0.750 (95% CI 0.19–0.99)	+0.750	—
Accuracy	0.781 (95% CI 0.60–0.91)	0.969 (95% CI 0.84–1.00)	+0.188	—
McNemar Exact Test	—	—	—	p = 0.032

Abbreviations: CI = confidence interval; PCI = percutaneous coronary intervention; RAG = retrieval-augmented generation

Note: Predicted PCI recommendation was defined as predicted iFR ≤ 0.89; operator management was defined as PCI performed versus no PCI during the index procedure (n = 32; 28 PCI / 4 no PCI).

Supplementary Table S7. Structured Qualitative Comparison of Reasoning Components

Domain	Specific Observed Terminology (No-RAG)	Specific Observed Terminology (RAG)	Observed Structural Difference
**Morphology descriptors**	"long diffuse atherosclerotic disease," "focal high-grade stenosis," "complex LAD disease"	Same descriptors present but followed by physiologic context	No-RAG escalates directly; RAG contextualizes
**Immediate PCI framing**	"Recommended intervention strategy (elective PCI)" appears early	PCI recommendation follows physiology framing	RAG delays escalation
**Bifurcation emphasis**	"Provisional bifurcation PCI strategy"	Bifurcation strategy framed within physiology-guided context	Strategy less reflexive
**Physiology terminology**	Limited	"Physiology-guided assessment," "Post-PCI iFR ≥0.95"	RAG explicitly references physiologic thresholds
**Trial references**	Sometimes present	"FAME trial," "ILUMIEN III"	RAG embeds trial evidence structurally
**Guideline references**	Present	"ACC/AHA/SCAI Guideline for Coronary Artery Revascularization"	RAG uses guideline as reasoning scaffold
**Uncertainty placement**	"Uncertainty flag" appears late	Uncertainty appears earlier in reasoning	RAG moderates early certainty
**Clinical syndrome framing**	Often omitted or secondary	"Chronic coronary syndrome," "Stable ischemic heart disease," "Elective PCI setting"	RAG anchors case in syndrome context

**Medication discussion**	"On DAPT (ASA + ticagrelor)"	Integrated with management and risk discussion	RAG integrates systemic context
**Renal considerations**	Rare	"contrast-associated AKI," "baseline risk," "monitor K/Cr"	RAG incorporates systemic physiologic risk

Abbreviations: ACC/AHA/SCAI = American College of Cardiology/American Heart Association/Society for Cardiovascular Angiography and Interventions; AKI = acute kidney injury; ASA = aspirin; Cr = creatinine; DAPT = dual antiplatelet therapy; FAME = Fractional Flow Reserve Versus Angiography for Multivessel Evaluation; iFR = instantaneous wave-free ratio ; ILUMIEN III: OPTIMIZE PCI = OPtical Coherence Tomography (OCT) Compared to Intravascular Ultrasound (IVUS) and Angiography to Guide Coronary Stent Implantation: a Multicenter RandomIZEd Trial in Percutaneous Coronary Intervention (PCI); K = potassium; LAD = left anterior descending artery; PCI = percutaneous coronary intervention; RAG = retrieval augmented generation

Supplementary Figure S1. Precision–Recall Curves for Ischemia Classification.

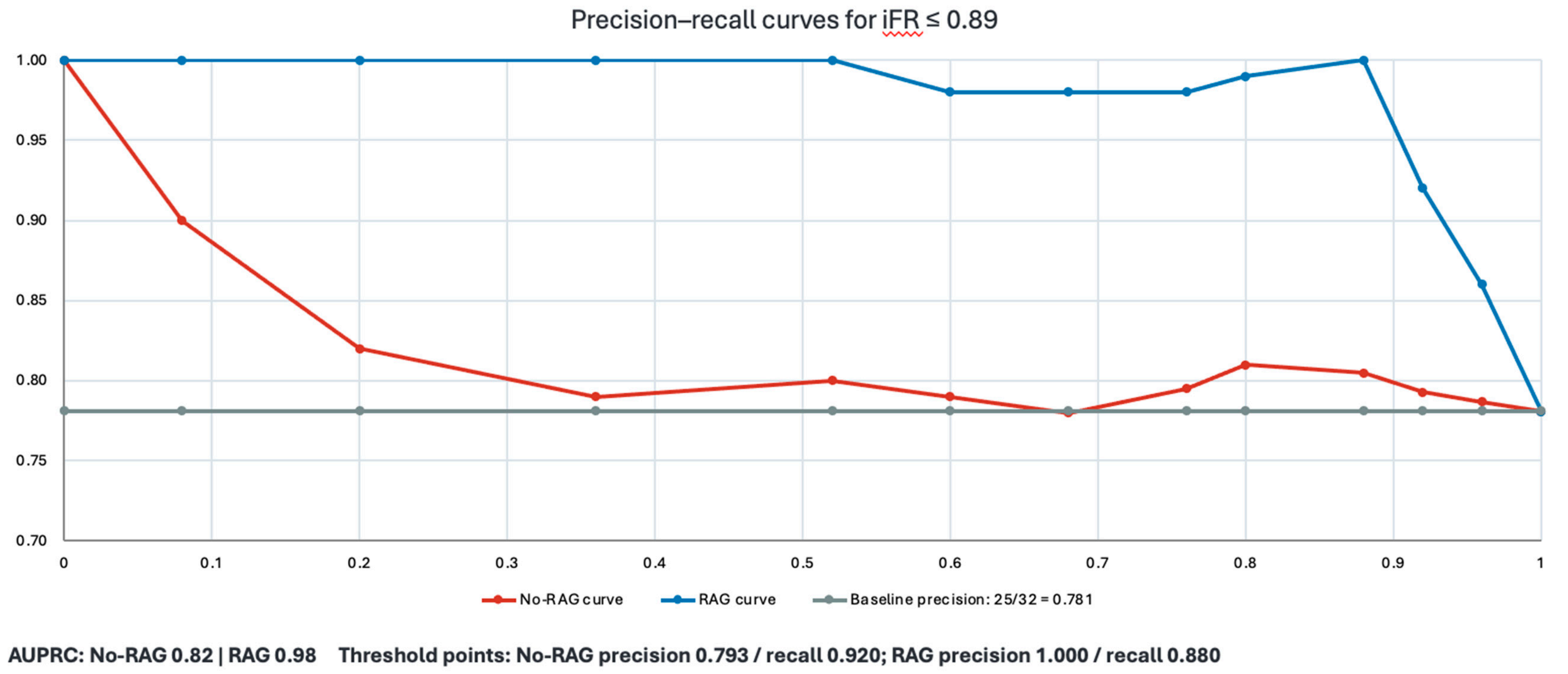

Caption: Precision–recall curves were generated by sweeping thresholds across case-level predicted iFR values, with lower predicted iFR treated as higher predicted likelihood of ischemia. The horizontal reference line indicates the cohort ischemia prevalence of 25/32, or 78.1%.
